# Purpura Hemorrhagica: Also Called Eruptic Fever, Petechial Fever, Anasarca, Etc.1Read before the Schuylkill Valley Veterinary Medical Association.

**Published:** 1895-01

**Authors:** J. W. Sallade


### PURPURA HEMORRHAGICA: ALSO CALLED ERUPTIC FEVER, PETECHIAL FEVER, ANASARCA, ETC.[1]

BY J. W. SALLADE, V. S.

THIS is a disease of a non-contagious, febrile character, of an intermittent nature. It is not a specific fever, but rather a secondary fever produced by dead material from suppurating surfaces and the oxidation and absorption of the colloid mass (a substance resembling glue or jelly) contained in the tissues, giving rise to a serious disease of the vasomotor system which governs the circulation in the smaller bloodvessels and capillaries. The capillaries of the skin and mucous membranes are especially implicated; they become congested with blood, which exudes through their walls, causing characteristic phenomena.

SYMPTOMS. The disease commences with symptoms which are very variable. In some instances the earliest symptoms may be pain and slight swelling in one or more legs, or a slight swelling in some other part of the body unaccompanied by pain, or again you may have your attention drawn to the fact that the animal is not doing quite so well—that he does not eat so well, that he appears stiff, or else is sluggish and is at a sort of standstill, is not convalescing so rapidly as he might, when, upon examination, you find small purple spots upon the Schneiderian membrane; without any other evidence this condition is proof positive of the existence of this trouble; but the appearance and local lesions are so unlike in gravity and location, and the constitutional phenomena so variable, that the original trouble is often masked. At first the pulse may be normal, and is seldom anything but normal, the temperature very little disturbed, the appetite good; but after two or three days the temperature rises to 102° or 103°, and often reaches 105°. The pulse now labors at 70 or 80 per minute, the appetite is variable, swellings appear in different parts of the body; these occur on the surface of the body, generally on the legs, lips, side of the head, on the forearm or under surface of the body. These swellings are seldom hot, somewhat sensitive, and are distinctly circumscribed from adjacent healthy tissues by a marked line. Thus the swelling in the hind leg ends abruptly above, as if a cord had been tied

[1] Read before the Schuylkill Valley Veterinary Medical Association.

around the leg. The swellings are due, as already stated, to a congested condition of the capillaries and small bloodvessels of the skin, owing to paralysis of the vasomotor system and an extravasation of blood into the areolar tissue, and the transudation of serum and feebly coagulable lymph. The swellings rapidly increase, the animal is rendered stiff by the swelling of the legs, and frequently stands rooted, as it were, to the same spot, disinclined to move; shows pain by lifting one leg and then the other; and the swelling may affect only one leg or it may affect all four at the same time, or any number of them; again, it often leaves one to appear in another, or leaves all to appear in the head, which often swells to an awkward and alarming size, the nose and lips swelling and the swelling extending back to the throat, causing closure of the nostrils, or else pressure on the larynx, which gives rise to difficult breathing, sometimes great dyspnœa, threatening and even causing suffocation. The small purple spots in the Schneiderian membrane gradually assume a brownish and frequently a black color, and coalesce, forming large blotches from which there occur frequent hemorrhages, the blood being very dark in color. The swelling of the head often interferes with the movements of the tongue and jaws, preventing the animal from eating, which increases the already great debility that exists. Œdema of the lungs, causing asphyxia, and colicky pains indicate that the bowels are attacked. I have had cases in which there was swelling or œdema of the throat and dyspnœa without any external swelling.

CAUSES. In the great majority of cases purpura occurs as a sequel of some debilitating disease, such as influenza, and its origin in most cases is directly traceable to bad ventilation or bad drainage. Overwork during convalescence from such disease, and while the nervous system is yet in a debilitated condition, is also an exciting cause. Exposure to cold and exposure to cold draughts when heated may cause it, but with proper ventilation and drainage these latter conditions more frequently cause pneumonia, pleurisy, etc. In the majority of cases the hygiene of the stable is directly responsible for this disease. Of course, the effete matter contained in the system, the condition of the blood and vasomotor system, or its action on the same, renders the animal prone to its development.

TERMINATIONS. Resolution. This is the most favorable as well as the most frequent termination, and may be looked for at the end of the third week, the animal being ready to go to light work after the first month. It is brought about by absorption of the effusion and profuse diuresis. Even when this takes place there is at times left some permanent induration.

METASTASIS. The dropsical swellings often disappear from one part of the body and reappear in another. This is a dreaded complication. As long as the swellings are confined to the external surfaces of the body, and do not involve the head and neck too much, no great danger may be apprehended; but when it suddenly disappears from the legs and under surfaces of the body, more especially during the first week or ten days, and the animal shows colicky pains or labored breathing, the case becomes apprehensive. Diarrhœa, the fecal matter tinged with blood, a suppressed, deep, hollow cough, a ropy bloody discharge from the nostrils, all indicate that the swelling has changed its position to the inside and that the horse is threatened with death from either asphyxia or enteritis, or perhaps effusion into some serous sac. In pulmonary œdema the parenchyma of the lungs is affected and the horse dies of asphyxia.

SUPPURATION. Suppuration ending frequently in gangrene, especially in small localized spots, which crack and slough, leaving ugly fissures and ulcers to heal. The exudated fluid exerts such a pressure on the tissues that they can no longer resist its force; sweating over the swollen parts of the skin then takes place, vesicles form upon the lower parts of the limbs, around the flexor surfaces of the joints; cracks and fissures appear, from which an unhealthy, fetid, amber-colored discharge issues; the skin sloughs, as do also the subjacent tissues, and the case terminates fatally by exhaustion.

PERITONITIS. Peritonitis may be produced either by the enteric œdema or by perforation of the wall of the viscera by gangrenous spots.

SEPTICÆMIA. Septicæmia is another result or termination of the disease which offers such a favorable field for its development. This is ushered in by severe rigors, sudden rise of temperature, great debility, and coma.

DEATH. Death may be produced by closure of the nostrils or the glottis, asphyxia, hemorrhage, enteritis, exhaustion, etc.

POST-MORTEM APPEARANCES. The post-mortem lesions are very simple. The capillaries are dilated, the connective tissue filled with a cinnamon-colored exudate, and the muscles separated by this jelly-like, colloid mass, which fills and stretches the meshes of the connective tissue until their web-like structure is expanded like a sponge; dark spots are found on the theca of the muscles, which when cut into are found to be but superficial, seldom extending deep into the substance of the muscles; the pleura, pericardium, endocardium, meninges of the brain and other serous membranes are covered with petechial spots; upon the external surfaces are found abscesses, ulcers, and gangrenous spots; if there was œdema of the lungs, these organs are distended, the bronchi and trachea filled with dark-colored extravasations, the mucous membrane studded with purple spots, etc.

PROGNOSIS. Prognosis depends upon the complications. Although the case looks formidable, with proper accommodations the same may be favorable, yet it is best to be on the safe side, and so for the first week or ten days be a little guarded in our prognosis.

TREATMENT. The great requisite in the successful treatment of this disease is to provide favorable surroundings. Hygiene plays a most important part; bad air, bad smells, bad drainage and poor ventilation, the chief causes of the trouble, engage our first attention. The mortality is great or small in proportion to the wisdom we exercise in this direction. I have treated a great many cases of purpura, and can safely say have lost but very few of them, although I have had them so weak and debilitated that it was necessary to raise and support them in the slings. My first endeavor is to secure proper ventilation—an open shed preferred, and comfortable clothing, pure water, and proper drainage. The next step is to regulate the disordered circulation and brace up the vasomotor system; to this end I administer twice a day the following: ℞.—Pulv. chlorate of potash, 1 ounce; spts. of turpentine, 1 ounce; spts. camphor, 2 drachms; ol. lini, 6 ounces; mix; this I give night and morning, and a dose of dilute nitric acid, say 1 drachm at noon. I now watch the case closely, observing the action of the medicine on the bowels and kidneys, and renew these doses as often as the condition of affairs will safely permit. The turpentine is given for its stimulant effect upon the heart and circulation, as well as for its warm diuretic properties. The chlorate of potash acts as a liberator of oxygen and an antiseptic. The camphor stimulates the vasomotor system and lessens the action of the pneumogastric, thus increasing the circulation and raising the arterial tension, while the oil acts as a laxative. The nitric acid I prefer to the sulphuric acid generally recommended, for the reason that it increases the secretions, while sulphuric acid lessens them, and is just as good a tonic. However, where an astringent is required to localize its action at a point remote from the stomach, as perhaps in hemorrhage from the kidneys or bowels, the sulphuric may be substituted. By this treatment absorption is promoted, the excretions increased, the circulation toned up, the vasomotor system stimulated; in fact, the entire system jared and belted up so that the weakened organs renew their functional activity and the products of decomposition are carried off. The acid I generally continue throughout the entire course of treatment. The same with the camphor. In cases where there is a good deal of soreness of the throat I give 1 drachm doses of tinct. of iron and 2 drachm doses of chlorate of potash in solution. I also employ, with the best of results, the bichromate of potash in nearly all of my cases, giving it both internally in minute doses several times a day and use it as a wash over the swollen parts in very weak solution. I find that it has a specially stimulating action on the lymphatic system.

EXTERNALLY. I do not believe in interfering much with the swollen parts more than has already been intimated. If the swelling becomes excessive about the head, bathing with a weak solution of the bichromate, 10 grains to a pint of water, is all that is necessary. Denuded surfaces, sloughs, etc., should be washed and disinfected. Tracheotomy may become necessary where mechanical asphyxia is threatened, but I never yet had a case where I deemed it necessary, although I had them gaping pretty loud for breath in my time. Where there is wheezing and slobbering or frothing at the mouth I find that a sharp blister of red iodide of mercury applied to the throat gives speedy relief. If, however, the dyspnœa is due to closure of the nostrils or closure of the glottis this alone may not avail. However, I am almost egotistic enough to assert that if called in time, and the veterinarian follows the general rules laid down in this humble effort, there will be no such serious complications. Upon the first sign of internal complications, such as pawing, labored breathing, etc., apply a mustard plaster over the entire chest and belly of the animal. The peripheral irritation set up by the sinapism draws the current of the circulation to the exterior, stimulates the nervous system, and prevents metastasis to the lungs and intestines.

HYGIENE. Hygiene, as already indicated, is a most important factor in the treatment of this disease. As regards food and water, I prefer to keep constantly before the animal a liberal supply of fresh pure water, changing it frequently, and allow any kind of food the animal will take and that the stomach is able to take care of; let it be clean and pure, but do not allow it to accumulate in the manger, for the horse will sicken at the very sight of it. Milk is very good food, and you may find it necessary to administer milk, egg-nog, whiskey, etc., as well as medicine per rectum. Always bear in mind that it is really, after all, food that gives permanent tone and strength to the tissues.

